# Potential of Antifungal Proteins (AFPs) to Control *Penicillium* Postharvest Fruit Decay

**DOI:** 10.3390/jof7060449

**Published:** 2021-06-04

**Authors:** Mónica Gandía, Anant Kakar, Moisés Giner-Llorca, Jeanett Holzknecht, Pedro Martínez-Culebras, László Galgóczy, Florentine Marx, Jose F. Marcos, Paloma Manzanares

**Affiliations:** 1 Food Biotechnology Department, Instituto de Agroquímica y Tecnología de Alimentos (IATA), Consejo Superior de Investigaciones Científicas (CSIC), Catedrático Agustín Escardino Benlloch 7, 46980 Valencia, Spain; mgandia@iata.csic.es (M.G.); mginer@iata.csic.es (M.G.-L.); pedro.martinez@uv.es (P.M.-C.); jmarcos@iata.csic.es (J.F.M.); 2 Departamento de Medicina Preventiva y Salud Pública, Ciencias de la Alimentación, Bromatología, Toxicología y Medicina Legal, Universitat de València, Vicente Andrès Estellès s/n, 46100 Valencia, Spain; 3 Biocenter, Institute of Molecular Biology, Medical University of Innsbruck, Innrain 80-82, 6020 Innsbruck, Austria; anant.kakar@gmail.com (A.K.); jeanett.holzknecht@i-med.ac.at (J.H.); florentine.marx@i-med.ac.at (F.M.); 4 Institute of Plant Biology, Biological Research Centre, Eötvös Loránd Research Network, Temesvári krt. 62, 6726 Szeged, Hungary; galgoczi.laszlo@brc.hu; 5 Department of Biotechnology, Faculty of Science and Informatics, University of Szeged, Közép fasor 52, 6726 Szeged, Hungary

**Keywords:** *Penicillium* decay, *Penicillium* *digitatum*, *Penicillium* *italicum*, *Penicillium* *expansum*, PAFB antifungal protein, postharvest protection

## Abstract

*Penicillium* phytopathogenic species provoke severe postharvest disease and economic losses. *Penicillium expansum* is the main pome fruit phytopathogen while *Penicillium digitatum* and *Penicillium italicum* cause citrus green and blue mold, respectively. Control strategies rely on the use of synthetic fungicides, but the appearance of resistant strains and safety concerns have led to the search for new antifungals. Here, the potential application of different antifungal proteins (AFPs) including the three *Penicillium chrysogenum* proteins (PAF, PAFB and PAFC), as well as the *Neosartorya fischeri* NFAP2 protein to control *Penicillium* decay, has been evaluated. PAFB was the most potent AFP against *P. digitatum*, *P. italicum* and *P. expansum*, PAFC and NFAP2 showed moderate antifungal activity, whereas PAF was the least active protein. In fruit protection assays, PAFB provoked a reduction of the incidence of infections caused by *P. digitatum* and *P. italicum* in oranges and by *P. expansum* in apples. A combination of AFPs did not result in an increase in the efficacy of disease control. In conclusion, this study expands the antifungal inhibition spectrum of the AFPs evaluated, and demonstrates that AFPs act in a species-specific manner. PAFB is a promising alternative compound to control *Penicillium* postharvest fruit decay.

## 1. Introduction

Postharvest decay caused by phytopathogenic fungi provokes major economic losses for the worldwide industry of fresh horticultural products [1]. Furthermore, some fungal phytopathogens are responsible for the contamination of fruits and derivative products through the production of mycotoxins that are detrimental to human health [2]. Species from the *Penicillium* genus cause severe postharvest fruit diseases even when postharvest technologies are applied. *Penicillium digitatum*, the cause of citrus green mold, and *Penicillium italicum*, the cause of citrus blue mold, are the main responsible agents of postharvest citrus losses worldwide, since more than 90% of citrus rots are produced by these two species [3]. *P. digitatum* commonly causes larger losses during commercialization due to its predominance at ambient temperatures, while *P. italicum* decay is higher in cold-stored citrus fruits because it is predominant at temperatures below 10 °C [4]. *Penicillium expansum*, a very aggressive cosmopolitan fungus, causes blue mold rot or soft rot on many economically important fruit and vegetable crops [5], and primarily causes the rot of stored apples and pears [6]. *P. expansum* is also of concern in fruit-based products because of its production of the mycotoxin patulin [7].

Currently, the most common method to control postharvest rot of fruit and vegetables is the application of synthetic fungicides. Despite their effectiveness, the continuous use of fungicides has resulted in the development of resistant fungal strains that compromise the success of the control strategy. Moreover, environmental contamination, strict regulatory reviews and safety demands from consumers have led to the search for new antifungal agents. Ideally, newly developed antimycotics should combine major aspects such as minimal impact on the environment, high efficacy, limited toxicity, and low costs of production [1].

Antifungal proteins (AFPs) secreted by filamentous ascomycetes offer great potential as new biofungicides [8,9]. AFPs are small, cationic, cysteine-rich proteins that fold into compact structures stabilized by disulphide bonds. They are highly stable to extreme pH, resistant to high temperature and proteolysis, and are able to inhibit the growth of opportunistic human, animal, plant and foodborne pathogenic fungi at micromolar concentrations [10,11,12]. Moreover, AFPs can be regarded as safe [13,14,15,16,17,18,19,20,21] and can be produced in efficient fungus- or plant-based biofactories [22,23].

Filamentous fungi, including pathogens, have a complex repertoire of AFPs, which differ in amino acid composition and sequence, and which can be grouped into different phylogenetic groups [24,25,26,27]. Some fungal genomes such as *Penicillium chrysogenum* and *Neosartorya* (*Aspergillus*) *fischeri* encode AFPs from different groups [24,25,26,27]. *P. chrysogenum* harbors three genes that code for PAF, one of the first identified and most studied AFPs [28], and the recently described PAFB [15,29] and PAFC [30]. Likewise, *N. fischeri* encodes for three phylogenetically distant AFPs, NFAP, NFBP and NFAP2 [26,27]. Although *P. chrysogenum* and *N. fischeri* AFPs have been functionally characterized and promise treatment alternatives to licensed antifungal drugs or biofungicides [20,21], there is a lack of information about their potential antifungal activity against phytopathogenic *Penicillium* species. Only PAF has been previously evaluated in vitro against *P. digitatum*, *P. italicum* and *P. expansum*, all of which were moderately resistant to the protein [14]. Phytopathogenic *Penicillium* genomes also encode AFPs. *P. expansum* genome encodes three phylogenetically distinct AFPs, PeAfpA, PeAfpB and PeAfpC, whereas *P. digitatum* and *P. italicum* only harbor one *afp* gene [24]. The *P. digitatum* protein, PdAfpB, was the first characterized protein from a fungal pathogen [14,24], while *P. italicum* AFP is yet to be experimentally described. PeAfpA and PdAfpB were characterized as highly active against *Penicillium* species, and remarkably as self-inhibitory proteins [13,14]. Moreover, PeAfpA efficiently protects against fungal infections caused by *P. digitatum* in oranges [13] and *P. expansum* in apples [31].

This study aims to evaluate the potential application of the recently described *P. chrysogenum* PAFB and PAFC and *N. fischeri* NFAP2 in postharvest fruit protection compared to the well-studied PAF and the highly active PdAfpB and PeAfpA. We characterize their in vitro antifungal profile against *P. digitatum*, *P. italicum* and *P. expansum*. In addition, we describe their effectiveness to control *Penicillium* decay in orange and apple fruits. Finally, the potential use of AFP combinations in fruit protection assays is discussed.

## 2. Materials and Methods

### 2.1. Strains, Media and Growth Conditions

**Figure 1:**
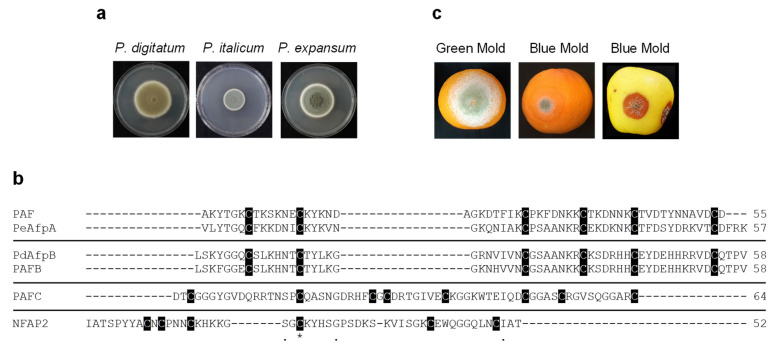
*Penicillium* species and antifungal proteins (AFPs) evaluated. (**a**) Representative images of growth on PDA plates of *P. digitatum* PHI-26 CECT 20796, *P. italicum* PHI-1 CECT 20909 and *P. expansum* CMP-1 CECT 20906 strains. (**b**) Amino acid sequence alignment of the tested AFPs. Phylogenetically different proteins are separated by a line. Conserved amino acids are represented with (*). Other conserved amino acids are represented with (.). Cysteine residues are shadowed in black. Amino acid alignment was performed using Clustal Omega (https://www.ebi.ac.uk/Tools/msa/clustalo/ (accessed on 28 November 2020)). The number of amino acids of proteins is also shown at the end of the amino acid sequence. (**c**) Images of infected orange fruits with *P. digitatum* (green mold) and *P. italicum* (blue mold) and infected apple fruits with *P. expansum* (blue mold) at 7 days post-inoculation (dpi).

For generation of conidia, fungi were cultured on Potato Dextrose Agar (PDA; Difco-BD Diagnostics, Sparks, MD, USA) plates for 5 (*P. chrysogenum* strains)–7 (*P. digitatum*, *P. italicum* and *P. expansum* strains) days at 25 °C. For antifungal assays, *P. digitatum* CECT 20796 (PHI26) [32], *P. italicum* CECT 20909 (PHI1) and *P. expansum* CECT 20906 (CMP-1) [33] strains were used. Representative images of fungal growth on PDA plates are shown in Figure 1a.

### 2.2. AFP Production and Purification

PAF, PAFB, PAFC, NFAP2 and PdAfpB were produced with the *P. chrysogenum*-based expression system under the regulation of the strong *paf* promoter [23] in either *P. chrysogenum* (PAF, PAFB, PAFC and NFAP2) [15,23,30,34] or *P. digitatum* (PdAfpB) [35]. Recombinant AFPs and PeAfpA from wild-type *P. expansum* CMP-1 strain [13] were purified following published procedures [14,15,23,30,34]. Briefly, strains were grown either in *P. chrysogenum* Minimal Medium (PcMM) (*P. chrysogenum* and *P. expansum*) or *P. digitatum* Minimal Medium (PdMM) [23] at 25 °C, and proteins purified from cell-free supernatants by one-step cation exchange chromatography. Protein concentrations were determined spectrophotometrically (A280) considering their respective molar extinction coefficients. The amino acid sequences of AFPs used and their physicochemical properties are shown in Figure 1b and Table 1.

### 2.3. Antifungal Assays

Susceptibility tests were carried out in 96-well, flat-bottom microtiter plates (Thermo Scientific, Waltham, MA, USA) as described before [15]. Briefly, 100 µL of conidia (1 × 10^4^ conidia/mL) in 10% (*v/v*) PDB were mixed with 100 µL of AFP prepared in serial twofold dilutions (in 10% (*v/v*) PDB) to reach final concentrations of 0–64 µM. Plates were statically incubated for 72 h at 25 °C. Growth was determined every 24 h by measuring the optical density (OD) of the fungal cultures at 620 nm using FLUOstar Omega plate spectrophotometer (BMG Labtech, Ortenberg, Germany), and the OD_620_ mean and standard deviation (SD) of three replicates were calculated. Minimum inhibitory concentration (MIC) was defined as the protein concentration that completely inhibited fungal growth ≥90% in all the independent experiments performed (*n* = 2–3).

### 2.4. Protection Assays against Fungal Infections Caused by Penicillium spp. in Fruits

Assays were conducted either with freshly harvested oranges (*Citrus sinensis* L. Osbeck cv Navel and Lanelate) or with apples (*Malus domestica* cv Golden Delicious) obtained from a local grocery. Fruits were surface sterilized by incubation for 5 min in a 5% commercial bleach solution and subsequent washing in distilled water three times, and air-dried. For protection assays, three replicates of five fruits were inoculated at four wounds around the equator with 5 µL of conidial suspensions (10^4^ conidia/mL for *P. digitatum* and *P. expansum*, and 2.5 × 10^4^ for *P. italicum*), which were pre-incubated for 24 h with 100 μg/mL of each AFP. Fruits were stored at 20 °C and 90% relative humidity. Each wound was scored daily for infection symptoms on consecutive days post-inoculation (dpi). Statistical analyses were performed using IBM SPSS Statistics v.26 to calculate one-way ANOVA and Tukey’s HSD test (*p* < 0.05). Representative images of infected *P. digitatum* and *P. italicum* orange fruits and *P. expansum* apple fruits are shown in Figure 1c.

## 3. Results

### 3.1. Antifungal Activity Assays

The three *P. chrysogenum* AFPs and *N. fischeri* NFAP2 were tested for their antifungal activity against the three main *Penicillium* species that cause severe postharvest fruit diseases (*P. digitatum*, *P. italicum* and *P. expansum*) (Figure 1a,c). Although previously characterized, PAF was included in the antifungal assays as an internal control for a better comparison among *P. chrysogenum* AFPs. Amino acid sequence alignment and physicochemical properties of the AFPs evaluated together with those of the highly active PeAfpA [13] and PdAfpB [14] are summarized in Figure 1b and Table 1.

Table 2 shows MIC values of each AFP against *P. digitatum*, *P. italicum* and *P. expansum*. PAFB was the most potent AFP against the three *Penicillium* species, with MIC values similar to those described for PeAfpA and PdAfpB (Table 2) [13,14]. NFAP2 showed a moderate antifungal activity against *P. digitatum* and *P. italicum*, although no MIC value for *P. expansum* could be determined at the highest concentration tested (64 μM). Similarly, PAFC completely inhibited the growth of *P. digitatum* and *P. italicum* with MIC values of 4 μM and 8 μM, respectively, whereas the MIC value for *P. expansum* was reached at 32 μM. As expected, the three *Penicillium* species were moderately resistant to PAF, confirming previous results [14].

### 3.2. PAFB Delays P. digitatum Infection in Orange Fruits

**Figure 2:**
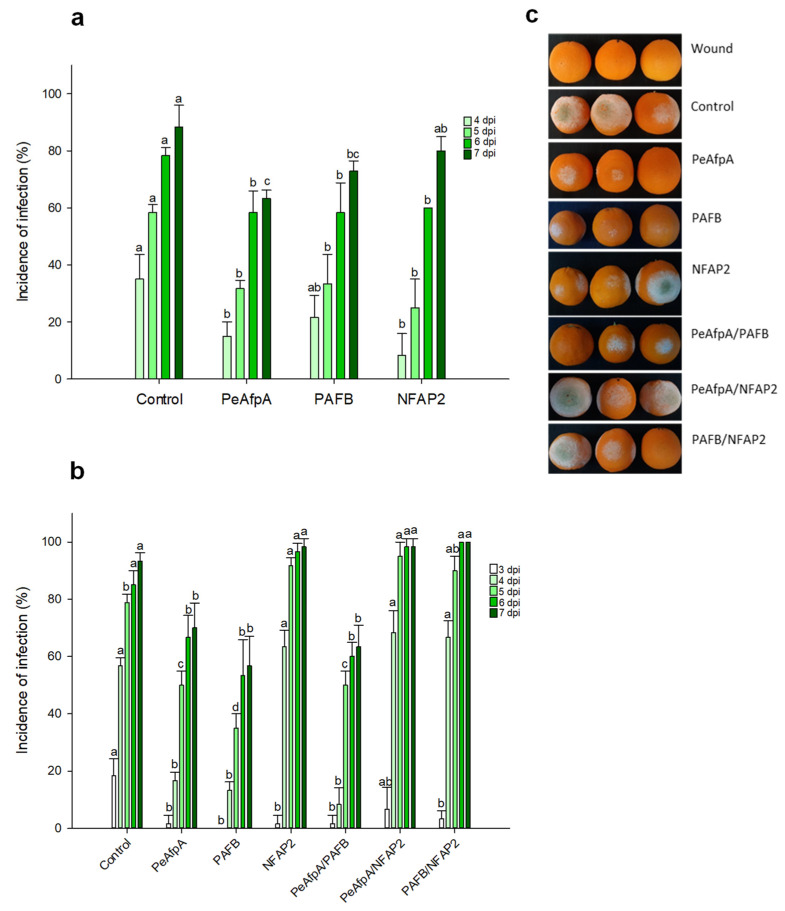
Effect of different antifungal proteins on the infection of orange fruits cv Navel by *P. digitatum*. (**a**) Effect of PeAfpA, PAFB and NFAP2 on the infection of orange fruits. (**b**) Effect of PeAfpA, PAFB, NFAP2 and combinations on the infection of orange fruits. Orange fruits were inoculated with 10^4^ conidia/mL of *P. digitatum* either alone (Control) or in the presence of 100 μg/mL of AFPs (corresponding to 15 μM PeAfpA, 16 μM PAFB and 18 μM NFAP2). Bars show the mean values of the percentage of infected wounds and standard deviation (SD) of three replicates of five oranges at 3, 4, 5, 6, and 7 dpi. Letters show significant differences among the treatments at each independent day (one-way ANOVA and Tukey’s HSD test, *p* < 0.05). (**c**) Representative images of treated oranges of (**b**) with AFPs and combinations at 7 dpi.

Based on the in vitro antifungal susceptibility test results, experiments were designed to evaluate the ability of the two most active proteins, PAFB and NFAP2, to control the green mold disease caused by *P. digitatum* infection to citrus fruit. PeAfpA, which had been previously described as effective in the control of the fungus in orange fruits [13], was included as an internal control. Figure 2a shows the effects of 100 μg/mL AFPs (corresponds to 15 µM PeAfpA, 16 µM PAFB and 18 µM NFAP2) in orange fruits from the Navel variety. The three proteins showed a slight control of *P. digitatum* infection. PeAfpA delayed fungal infection throughout the experiment; PAFB did not affect fungal growth at 4 dpi, although it significantly decreased the incidence of infection at 5, 6 and 7 dpi, while NFAP2 failed to control *P. digitatum* at 7 dpi. The average efficacy at 7 dpi was around 30% and 20% of disease reduction for PeAfpA and PAFB, respectively. In order to confirm the effect of AFPs, a second experiment with AFPs in individual and combined treatments was carried out (Figure 2b). Both PeAfpA and PAFB treatments resulted in a delay of fungal infection, although the average efficacy of disease reduction at 7 dpi of PeAfpA (25%) and PAFB (40%) varied with respect to that of Figure 2a. Regarding NFAP2, the effect was only statistically significant at 3 dpi. With respect to AFP combinations, the combined effect of PeAfpA and PAFB was not different to that caused by individual proteins, suggesting no additive or synergistic effects. Furthermore, combinations of either PeAfpA or PAFB with NFAP2 did not result in any protective effect. Figure 2c shows representative images of AFP-treated oranges at 7 dpi.

### 3.3. PAFB Delays P. italicum Infection in Orange Fruits

**Figure 3:**
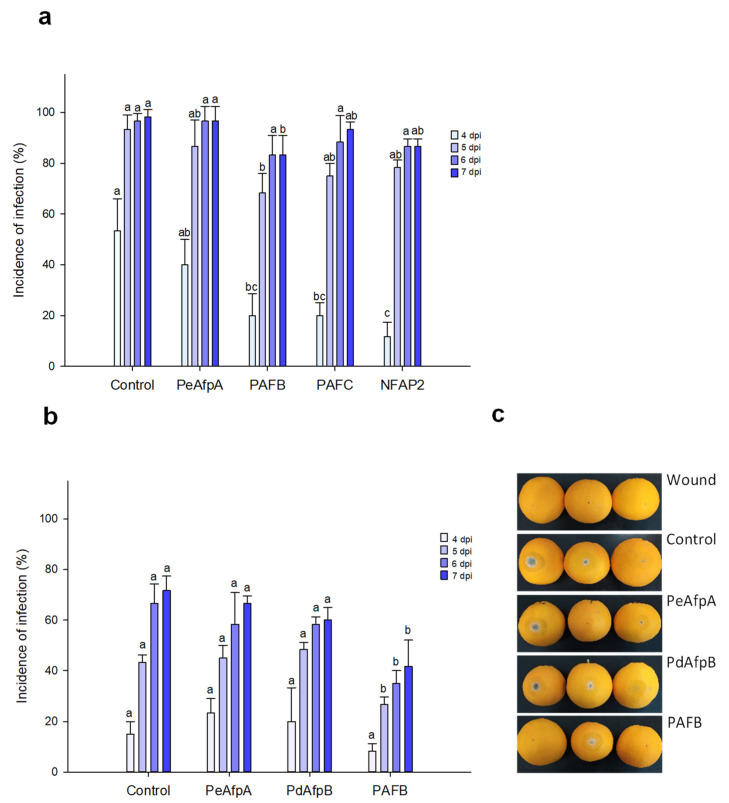
Effect of different antifungal proteins on the infection of orange fruits by *P. italicum*. (**a**) Effect of PeAfpA, PAFB, PAFC and NFAP2 on the infection of orange fruits cv. Navel. (**b**) Effect of PeAfpA, PdAfpB and PAFB on the infection of orange fruits cv. Lanelate. Orange fruits were inoculated with 2.5 × 10^4^ conidia/mL of *P. italicum* either alone (Control) or in the presence of 100 μg/mL of AFPs (corresponding to 15 μM PeAfpA, PAFC and PdAfpB, 16 μM PAFB and 18 μM NFAP2). Bars show the mean values of the percentage of infected wounds and standard deviation (SD) of three replicates of five oranges at 4, 5, 6 and 7 dpi. Letters show significant differences among the treatments at each independent day (one-way ANOVA and Tukey’s HSD test, *p* < 0.05). (**c**) Representative images of treated oranges of (**b**) with AFPs at 6 dpi.

Based on the individual MIC values against *P. italicum*, PAFB, PAFC and NFAP2 were selected to control the blue mold disease in orange fruits. PeAfpA, with a MIC value of 0.3 μM against this fungus (Table 2) [13], was included in the study since its in vivo effect has not been previously reported. Figure 3a shows the effect of the four proteins at 100 µg/mL (corresponds to 15 µM PeAfpA, PAFC and PdAfpB, 16 µM PAFB and 18 µM NFAP2). Only PAFB showed a slight reduction in the incidence of infection compared to the control with no AFP treatment. The PAFB average efficacy of blue mold reduction along the experiment varied from 60% at 4 dpi to 15% at 7 dpi. With the aim of confirming the efficacy of PAFB, a second infection experiment was designed including PdAfpB, another class B protein, whose MIC value against *P. italicum* was determined to be 0.3 μM (Table 2) [14]. Figure 3b shows the effect of both class B AFPs and PeAfpA on orange fruit infections from Lanelate variety. PAFB controlled the experimental *P. italicum* infection from 5 to 7 dpi while neither PdAfpB nor PeAfpA exhibited any infection inhibitory effect. Figure 3c shows representative images of AFP-treated Lanelate oranges at 6 dpi, where the average efficacy of PAFB was 50% disease reduction. At the end of the experiment (7 dpi), the average efficacy of PAFB treatment was 40% disease reduction.

### 3.4. PAFB Delays P. expansum Infection in Apple Fruits

**Figure 4:**
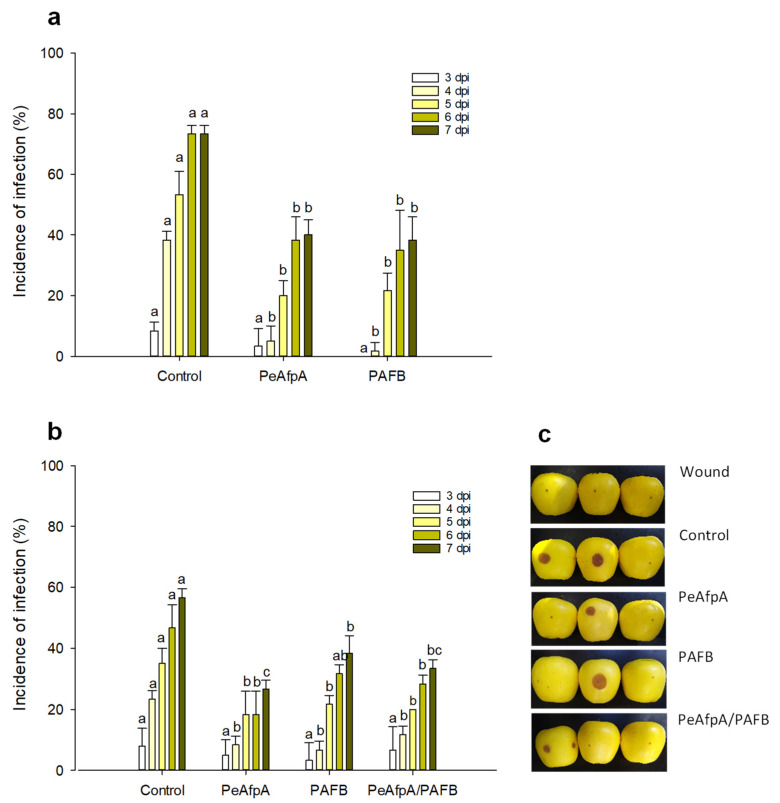
Effect of different antifungal proteins on the infection of apple fruits cv Golden by *P. expansum*. (**a**) Effect of PeAfpA and PAFB on the infection of apple fruits. (**b**) Effect of PeAfpA, PAFB and PeAfpA/PAFB on the infection of apple fruits. Apple fruits were inoculated with 10^4^ conidia/mL of *P. expansum* either alone (Control) or in the presence of 100 μg/mL of AFPs (corresponding to 15 μM PeAfpA and 16 μM PAFB). Bars show the mean values of the percentage of infected wounds and standard deviation (SD) of three replicates of five apples at 3, 4, 5, 6, and 7 dpi. Letters show significant differences among the treatments at each independent day (One-way ANOVA and Tukey’s HSD test, *p* < 0.05). (**c**) Representative images of treated apples of (**b**) with AFPs and combinations at 7 dpi.

Previous in vivo experiments indicated that PeAfpA efficiently protected apple fruits against *P. expansum* infections [31]. Here, we conducted apple inoculation experiments to assess the effectiveness of the most active AFP in vitro, PAFB, in comparison to PeAfpA. Figure 4a shows the effect of both proteins at 100 µg/mL (corresponds to 15 µM PeAfpA and 16 µM PAFB) on the *P. expansum* infection of Golden Delicious apples. Both PeAfpA and PAFB controlled the infection with similar efficacy (Figure 4a). At 7 dpi, disease reductions close to 50% were observed. To confirm the control of *P. expansum* infection by PAFB treatment, a second independent experiment was accomplished (Figure 4b). PAFB exerted a significant protective effect, although the efficacy after 7 dpi was lower (32% of disease reduction) than that observed in Figure 4a. Combination of PAFB and PeAfpA at 15 μM each did not further improve the effect caused by the individual treatments.

## 4. Discussion

Currently, *Penicillium* postharvest decay control strategies rely on the use of synthetic fungicides such as imazalil, sodium ortho-phenylphenate or thiabendazole, although extensive research for the development of new alternatives is being conducted [3,5]. Natural alternative methods include plant extracts, natural antifungal edible coatings and antifungal peptides and proteins [1] as those evaluated in this work.

In the present study, we describe the potential of several phylogenetically different AFPs as new agents for *Penicillium* decay control. The AFPs tested were PAF, PAFB and PAFC from *P. chrysogenum*, and NFAP2 from *N. fischeri*. Although antifungal activity of the four AFPs was previously tested against different microorganisms [15,27,30,34,36], the three main *Penicillium* species that cause significant economic losses after harvest, *P. digitatum*, *P. italicum* and *P. expansum*, were not included in those studies. The AFPs evaluated differ in amino acid composition, primary structure and physicochemical properties (Figure 1 and Table 1), which might explain the different in vitro antifungal profiles and in vivo efficacy in fruit inoculation experiments observed in this study.

PAFB displayed the highest antifungal potency in in vitro assays with MIC values in the range of 0.12–0.25 μM, comparable to those of PeAfpA (0.15–0.3 μM) [13] and PdAfpB (0.3–0.6 μM) [14]. PAFB also exhibits growth inhibitory activity against human pathogenic fungi such as *Aspergillus fumigatus*, *Trichophyton* spp. and *Candida* spp., and against the fungal model organisms *Neurospora crassa* and *Saccharomyces cerevisiae* [15]. Remarkably, these latter species were similarly sensitive towards PAFB and PAF with MIC values in the range 0.25–4 μM [15,25], while our results show that the three phytopathogenic *Penicillium* species tested were much more resistant to PAF (MIC values in the range 16–32 μM) than to PAFB. These results show that AFPs act in a species-specific manner.

PAFC and NFAP2 showed a moderate antifungal activity against *P. digitatum* and *P. italicum*, whereas the MIC values against *P. expansum* were much higher. The in vitro growth inhibitory activity of PAFC against opportunistic human pathogenic members of the *Candida* genus has been recently described [30]. Remarkably, PAFC shares 83% amino acid identity with the *P. expansum* class C protein, PeAfpC, which did not show any antifungal activity against the three *Penicillium* species tested [13]. Only recently, the antifungal activity of PeAfpC against several species of the genus *Byssochlamys* has been reported [37]. Regarding NFAP2, it was characterized having a unique high anti-yeast activity whereas it was ineffective against filamentous fungi [27]. Here, our results extend the antifungal spectrum of both PAFC and NFAP2 to filamentous fungi of the *Penicillium* genus.

The AFP from *Aspergillus giganteus* was the first experimentally tested ascomycetous antifungal protein to control plant and postharvest diseases in vivo. *A. giganteus* AFP has been reported to successfully control postharvest decay caused by *Magnaporthe oryzae* in rice [38], *Fusarium oxysporum* in tomato plant seeds [39] and the infection caused by *Alternaria alternata* in banana [40]. Here, based on the in vitro inhibitory efficacy, we selected different AFPs to evaluate their potential in the control of *Penicillium* decay using three pathosystems, *P. digitatum*-orange fruit, *P. italicum*-orange fruit and *P. expansum*-apple fruit. PAFB provoked disease reductions in orange and apple fruits, although with moderate efficacy. In agreement to that observed in the in vitro assays, the PAFB effect in protection experiments was equivalent to that observed with PeAfpA in the side-by-side experiments conducted. We showed previously that PeAfpA exerts protection against *P. digitatum* in oranges and against *P. expansum* in apples at concentrations of 0.15–15 µM, although variations in the percentage of disease reduction among experiments, as those observed here, were reported [13,31]. PAFB was also effective in delaying *P. italicum* infection to Navel and Lanelate oranges while PeAfpA did not show any effect. This is the first time that PeAfpA is evaluated in the pathosystem *P. italicum*-orange fruit, and despite its potency against the fungus in in vitro experiments [13], no protection effect was observed in any of the orange varieties tested. Discrepancies between in vitro and in vivo experiments were also found for PAFC, NFAP2 and PdAfpB, since none of them were able to efficiently protect orange fruits despite their in vitro determined MIC values against *P. digitatum* and *P. italicum* (Table 2). We previously described that PAF, but not the rationally designed variant PAF^opt^, was able to inhibit *Botrytis cinerea* infection in tomato plant leaves, although both proteins inhibited *B. cinerea* growth in vitro [20]. In in vivo experiments additional factors that are absent in or differ from in vitro assays impact the antifungal potential of AFPs, e.g., fruit-specific substrates may influence fungal growth and AFP susceptibility, or compounds present in orange peels and apple skins may interfere with the AFP activity. Our study emphasizes the need for in vivo protection assays, as those described here, to evaluate the feasibility of AFPs in postharvest control.

PAFB is orthologous to PgAFP identified in the supernatant of *P. chrysogenum* strain RP42C, originally isolated from dry-cured ham [41]. PgAFP exhibits potent inhibitory activity against the main mycotoxin-producing species of *Aspergillus* and *Penicillium* of concern for dry-ripened foods and it efficiently reduces counts of *Aspergillus flavus* and *Penicillium restrictum* inoculated on a dry fermented sausage [42]. Recently, the effect of PgAFP against *P. expansum* and *P. digitatum* growth on fruits has been evaluated [43]. Although in vitro growth inhibition was shown, no inhibitory effect was observed in oranges from Navelina and Lanelate varieties or in Golden Delicious apples. However, a protective effect was found in apples from the Royal Gala variety [43]. It should be mentioned that it is difficult to compare inter-laboratory in vivo experiments mainly due to different protocols of AFP application and fungal inoculation. Moreover, the effects of the fungal strain as well as of the fruit variety should be considered.

Results reported here for AFPs only show a delay in disease progression, far from the level of protection attributed to chemical fungicides. However, the conditions of our controlled inoculation experiments should be considered. AFPs are point-inoculated and mycelia growing out from the inoculation site (where the protein is absent) might contribute to disease incidence, while fungicides are commercially applied onto the entire fruit surface. In addition, the inoculum dose used in protection assays is aggressive since it renders around 80–100% of infection at 6–7 dpi. Interestingly, when fungicides such as imazalil and thiabendazole are applied in parallel assays with antifungal peptides under similar conditions as those described here, fungicides performed similarly and the average efficacy of disease reduction was not significantly different to that provoked by AFPs or antifungal peptides [44,45]. Further experiments mimicking commercial conditions of fungicide application are necessary to confirm the feasibility of AFPs in postharvest protection.

To reach the level of efficacy provided by conventional fungicides, combinations of alternative approaches of the same or different nature have been proposed [1]. Here, we have combined PeAfpA and PAFB, the two active AFPs in fruit protection experiments, to evaluate a potential additive or synergistic effect. However, the combination of both proteins in the control of *P. digitatum* infection in orange fruits (Figure 2) and *P. expansum* infection in apples (Figure 4), respectively, did not result in a synergistic effect and not even an additive effect was observed. To the best of our knowledge, this is the first time that a combination of AFPs has been reported in in vivo experiments, although under the conditions tested it did not improve the efficacy observed with the individual treatments. Notably, when either PAFB or PeAfpA were combined with NFAP2 (Figure 2c), the latter counteracted the effect of both proteins, suggesting a negative interaction between these AFPs. Nevertheless, optimization of concentrations and ratios at which AFPs should be combined require further research. In addition, a precise understanding of the mechanism of synergistic interaction of antimicrobials is necessary for the design of combined approaches. In this context, the mechanism of antifungal action of AFPs, as described for PAF, PAFB and PdAfpB, is complex and regulated [25,46]. These three proteins have an energy-dependent cell-penetrating mode of action followed by a series of intracellular regulated actions that end with cell collapse [15,25,46,47]. The PAFC mode of action also requires PAFC uptake and cytoplasmic localization before plasma permeabilization occurs [30]. In contrast, this mechanism differs from that of NFAP2, whose cell-killing activity seems connected to its pore-forming ability in the cell membrane [16]. Although the mechanism of PeAfpA is still unknown, our data suggest that the combination of two AFPs with different mechanisms of action as those of PAFB and NFAP2 do not result in an increase in the efficacy. Further studies are necessary to determine the PeAfpA mode of action as well as to unravel the potential interactions among AFPs. Combinations of AFPs with chemical fungicides or physical treatments are in progress.

## 5. Conclusions

This study provides additional knowledge about the three *P. chrysogenum* AFPs and *N. fischeri* NFAP2, which show a species-specific inhibition spectrum against the main *Penicillium* species causing postharvest decay. PAFB, the most active antifungal protein in in vitro susceptibility tests, delays *P. digitatum*, *P. italicum* and *P. expansum* infection in orange and apple fruits. Future efforts are currently directed to optimize the efficacy of PAFB through combinations with different postharvest control strategies.

## Figures and Tables

**Table 1:** Predicted physicochemical properties of mature antifungal proteins used in this work.

Protein	Amino Acids (aa)	Molecular Weight (Da) ^a^	Number of Cysteines	pI ^b^	GRAVY ^c^	Charge at pH 7 ^d^
PAF	55	6242	6	8.93	−1.374	+4.7
PeAfpA	57	6618	6	9.48	−1.081	+8.7
PdAfpB	58	6570	6	9.06	−1.000	+5.9
PAFB	58	6500	6	8.83	−1.031	+5.2
PAFC	64	6630	8	7.71	−0.767	+0.9
NFAP2	52	5600	6	9.01	−0.731	+5.2

^a^ Molecular weights of the proteins were determined experimentally [13,14,15,23,25,27,30,34]. ^b^ Theoretical isoelectric point (pI) of all the proteins were calculated with the Compute pI/Mw and ProtParam tools of the ExPASy Proteomics Server (https://www.expasy.org/ (accessed on 28 November 2020)). ^c^ The Grand Average of Hydropathy (GRAVY) value of different proteins were determined with GRAVY calculator (www.gravy-calculator.de (accessed on 28 November 2020)). ^d^ The charge at pH 7 was determined with ProteinCalculator v3.4 (www.protcalc.sourceforge.net (accessed on 28 November 2020)).

**Table 2:** In vitro minimum inhibitory concentrations (MICs) of AFPs against *Penicillium* species ^1^.

	*P. digitatum*	*P. italicum*	*P. expansum*	Reference
PAF	16 (100)	32 (200)	16 (100)	this work
PAFB	0.16 (1.0)	0.25 (1.63)	0.12 (0.78)	this work
PAFC	4 (26.5)	8 (53.0)	32 (212)	this work
NFAP2	2 (11.1)	1 (5.5)	>64 (>356)	this work
PeAfpA	0.15 (1.0)	0.3 (2.0)	0.3 (2.0)	[13]
PdAfpB	0.6 (4)	0.3 (2.0)	0.6 (4)	[14]

^1^ MICs are given in μM (μg/mL in parentheses) and were determined after 44 h of incubation at 25 °C.

## Data Availability

Not applicable.

## References

[1] Palou L., Ali A., Fallik E., Romanazzi G. (2016). GRAS, plant- and animal-derived compounds as alternatives to conventional fungicides for the control of postharvest diseases of fresh horticultural produce. Postharvest Biol. Technol..

[2] Marin S., Ramos A.J., Cano-Sancho G., Sanchis V. (2013). Mycotoxins: Occurrence, toxicology, and exposure assessment. Food Chem. Toxicol..

[3] Palou L., Bautista-Baños S. (2014). Penicillium digitatum, *Penicillium italicum* (Green Mold, Blue Mold). Postharvest Decay: Control Strategies.

[4] Plaza P., Usall J., Torres R., Lamarca N., Asensio A., Viñas I. (2003). Control of green and blue mould by curing on oranges during ambient and cold storage. Postharvest Biol. Technol..

[5] Errampalli D., Bautista-Banos S. (2014). Penicillium expansum (blue mold). Postharvest Decay. Control Strategies.

[6] Turechek W.W., Naqvi S.A.M.H. (2004). Apple diseases and their management. Diseases of Fruits and Vegetables Volume I: Diagnosis and Management.

[7] Tannous J., Keller N.P., Atoui A., El Khoury A., Lteif R., Oswald I.P., Puel O. (2018). Secondary metabolism in *Penicillium expansum*: Emphasis on recent advances in patulin research. Crit. Rev. Food Sci. Nutr..

[8] Galgóczy L., Marx F. (2019). Do Antimicrobial Proteins contribute to overcoming the hidden antifungal crisis at the dawn of a post-antibiotic era?. Microorganisms.

[9] Marcos J.F., Muñoz A., Pérez-Payá E., Misra S., López-García B. (2008). Identification and rational design of novel antimicrobial peptides for plant protection. Annu. Rev. Phytopathol..

[10] Delgado J., Owens R.A., Doyle S., Asensio M.A., Núñez F. (2016). Antifungal proteins from moulds: Analytical tools and potential application to dry-ripened foods. Appl. Microbiol. Biotechnol..

[11] Hegedüs N., Marx F. (2013). Antifungal proteins: More than antimicrobials?. Fungal Biol. Rev..

[12] Marx F., Binder U., Leiter É., Pócsi I. (2008). The *Penicillium chrysogenum* antifungal protein PAF, a promising tool for the development of new antifungal therapies and fungal cell biology studies. Cell. Mol. Life Sci..

[13] Garrigues S., Gandía M., Castillo L., Coca M., Marx F., Marcos J.F., Manzanares P. (2018). Three Antifungal Proteins From *Penicillium expansum*: Different Patterns of Production and Antifungal Activity. Front. Microbiol..

[14] Garrigues S., Gandía M., Popa C., Borics A., Marx F., Coca M., Marcos J.F., Manzanares P. (2017). Efficient production and characterization of the novel and highly active antifungal protein AfpB from *Penicillium digitatum*. Sci. Rep..

[15] Huber A., Hajdu D., Bratschun-Khan D., Gáspári Z., Varbanov M., Philippot S., Fizil Á., Czajlik A., Kele Z., Sonderegger C. (2018). New antimicrobial potential and structural properties of PAFB: A cationic, cysteine-rich protein from *Penicillium chrysogenum* Q176. Sci. Rep..

[16] Kovács R., Holzknecht J., Hargitai Z., Papp C., Farkas A., Borics A., Tóth L., Váradi G., Tóth G.K., Kovács I. (2019). *In vivo* applicability of *Neosartorya fischeri* antifungal protein 2 (NFAP2) in treatment of vulvovaginal candidiasis. Antimicrob. Agents Chemother..

[17] Palicz Z., Jenes Á., Gáll T., Miszti-Blasius K., Kollár S., Kovács I., Emri M., Márián T., Leiter É., Pócsi I. (2013). *In vivo* application of a small molecular weight antifungal protein of *Penicillium chrysogenum* (PAF). Toxicol. Appl. Pharmacol..

[18] Szappanos H., Szigeti G.P., Pal B., Rusznak Z., Szucs G., Rajnavolgyi E., Balla J., Balla G., Nagy E., Leiter E. (2006). The antifungal protein AFP secreted by *Aspergillus giganteus* does not cause detrimental effects on certain mammalian cells. Peptides.

[19] Szappanos H., Szigeti G.P., Pál B., Rusznák Z., Szűcs G., Rajnavölgyi É., Balla J., Balla G., Nagy E., Leiter É. (2005). The *Penicillium chrysogenum*-derived antifungal peptide shows no toxic effects on mammalian cells in the intended therapeutic concentration. Naunyn-Schmiedeberg’s Arch. Pharmacol..

[20] Tóth L., Boros E., Poor P., Ordog A., Kele Z., Varadi G., Holzknecht J., Bratschun-Khan D., Nagy I., Toth G.K. (2020). The potential use of the *Penicillium chrysogenum* antifungal protein PAF, the designed variant PAF^opt^ and its γ-core peptide Pγ^opt^ in plant protection. Microb. Biotechnol..

[21] Tóth L., Váradi G., Boros É., Borics A., Ficze H., Nagy I., Tóth G.K., Rákhely G., Marx F., Galgóczy L. (2020). Biofungicidal Potential of *Neosartorya* (*Aspergillus*) *fischeri* Antifungal Protein NFAP and Novel Synthetic γ-Core Peptides. Front. Microbiol..

[22] Shi X., Cordero T., Garrigues S., Marcos J.F., Daròs J.A., Coca M. (2019). Efficient production of antifungal proteins in plants using a new transient expression vector derived from tobacco mosaic virus. Plant Biotechnol. J..

[23] Sonderegger C., Galgóczy L., Garrigues S., Fizil Á., Borics A., Manzanares P., Hegedüs N., Huber A., Marcos J.F., Batta G. (2016). A *Penicillium chrysogenum*-based expression system for the production of small, cysteine-rich antifungal proteins for structural and functional analyses. Microb. Cell Fact..

[24] Garrigues S., Gandía M., Marcos J. (2016). Occurrence and function of fungal antifungal proteins: A case study of the citrus postharvest pathogen *Penicillium digitatum*. Appl. Microbiol. Biotechnol..

[25] Huber A., Galgóczy L., Váradi G., Holzknecht J., Kakar A., Malanovic N., Leber R., Koch J., Keller M.A., Batta G. (2020). Two small, cysteine-rich and cationic antifungal proteins from *Penicillium chrysogenum*: A comparative study of PAF and PAFB. Biochim. Biophys. Acta Biomembr..

[26] Sonderegger C., Váradi G., Galgóczy L., Kocsubé S., Posch W., Borics A., Dubrac S., Tóth G.K., Wilflingseder D., Marx F. (2018). The Evolutionary Conserved γ-Core Motif Influences the Anti-Candida Activity of the *Penicillium chrysogenum* Antifungal Protein PAF. Front. Microbiol..

[27] Tóth L., Kele Z., Borics A., Nagy L.G., Váradi G., Virágh M., Takó M., Vágvölgyi C., Galgóczy L. (2016). NFAP2, a novel cysteine-rich anti-yeast protein from *Neosartorya fischeri* NRRL 181: Isolation and characterization. AMB Express.

[28] Marx F., Haas H., Reindl M., Stoffler G., Lottspeich F., Redl B. (1995). Cloning, structural organization and regulation of expression of the *Penicillium chrysogenum paf* gene encoding an abundantly secreted protein with antifungal activity. Gene.

[29] Huber A., Lerchster H., Marx F. (2019). Nutrient Excess Triggers the Expression of the *Penicillium chrysogenum* Antifungal Protein PAFB. Microorganisms.

[30] Holzknecht J., Kühbacher A., Papp C., Farkas A., Váradi G., Marcos J.F., Manzanares P., Tóth G.K., Galgóczy L., Marx F. (2020). The *Penicillium chrysogenum* Q176 Antimicrobial Protein PAFC Effectively Inhibits the Growth of the Opportunistic Human Pathogen *Candida albicans*. J. Fungi.

[31] Gandía M., Monge A., Garrigues S., Orozco H., Giner-Llorca M., Marcos J.F., Manzanares P. (2020). Novel insights in the production, activity and protective effect of *Penicillium expansum* antifungal proteins. Int. J. Biol. Macromol..

[32] Marcet-Houben M., Ballester A.R., de la Fuente B., Harries E., Marcos J.F., González-Candelas L., Gabaldón T. (2012). Genome sequence of the necrotrophic fungus *Penicillium digitatum*, the main postharvest pathogen of citrus. BMC Genom..

[33] Ballester A.-R., Marcet-Houben M., Levin E., Sela N., Selma-Lázaro C., Carmona L., Wisniewski M., Droby S., González-Candelas L., Gabaldón T. (2015). Genome, transcriptome, and functional analyses of *Penicillium expansum* provide new insights into secondary metabolism and pathogenicity. Mol. Plant-Microbe Interact..

[34] Tóth L., Váradi G., Borics A., Batta G., Kele Z., Vendrinszky Á., Tóth R., Ficze H., Tóth G.K., Vágvölgyi C. (2018). Anti-Candidal Activity and Functional Mapping of Recombinant and Synthetic *Neosartorya fischeri* Antifungal Protein 2 (NFAP2). Front. Microbiol..

[35] Hernanz-Koers M., Gandía M., Garrigues S., Manzanares P., Yenush L., Orzaez D., Marcos J.F. (2018). FungalBraid: A GoldenBraid-based modular cloning platform for the assembly and exchange of DNA elements tailored to fungal synthetic biology. Fungal Genet. Biol..

[36] Kaiserer L., Oberparleiter C., Weiler-Gorz R., Burgstaller W., Leiter E., Marx F. (2003). Characterization of the *Penicillium chrysogenum* antifungal protein PAF. Arch. Microbiol..

[37] Martínez-Culebras P.V., Gandía M., Boronat A., Marcos J.F., Manzanares P. (2021). Differential susceptibility of mycotoxin-producing fungi to distinct antifungal proteins (AFPs). Food Microbiol..

[38] Vila L., Lacadena V., Fontanet P., del Pozo A.M., Segundo B.S. (2001). A protein from the mold *Aspergillus giganteus* is a potent inhibitor of fungal plant pathogens. Mol. Plant-Microbe Interact..

[39] Theis T., Marx F., Salvenmoser W., Stahl U., Meyer V. (2005). New insights into the target site and mode of action of the antifungal protein of *Aspergillus giganteus*. Res. Microbiol..

[40] Barakat H. (2014). Bio-Control of *Alternaria alternata* during Banana Storage by Purified AFP Using Isoelectric Focusing Technique. Food Nutr. Sci..

[41] Rodriguez-Martín A., Acosta R., Liddell S., Nunez F., Benito M.J., Asensio M.A. (2010). Characterization of the novel antifungal protein PgAFP and the encoding gene of *Penicillium chrysogenum*. Peptides.

[42] Delgado J., Acosta R., Rodriguez-Martin A., Bermudez E., Nunez F., Asensio M.A. (2015). Growth inhibition and stability of PgAFP from *Penicillium chrysogenum* against fungi common on dry-ripened meat products. Int. J. Food Microbiol..

[43] Delgado J., Ballester A.-R., Núñez F., González-Candelas L. (2019). Evaluation of the activity of the antifungal PgAFP protein and its producer mould against *Penicillium* spp postharvest pathogens of citrus and pome fruits. Food Microbiol..

[44] Badosa E., Ferre R., Frances J., Bardaji E., Feliu L., Planas M., Montesinos E. (2009). Sporicidal Activity of Synthetic Antifungal Undecapeptides and Control of *Penicillium* Rot of Apples. Appl. Environ. Microbiol..

[45] López-García B., Veyrat A., Pérez-Payá E., González-Candelas L., Marcos J.F. (2003). Comparison of the activity of antifungal hexapeptides and the fungicides thiabendazole and imazalil against postharvest fungal pathogens. Int. J. Food Microbiol..

[46] Bugeda A., Garrigues S., Gandía M., Manzanares P., Marcos J.F., Coca M. (2020). The antifungal protein AfpB induces regulated cell death in its parental fungus *Penicillium digitatum*. mSphere.

[47] Leiter E., Szappanos H., Oberparleiter C., Kaiserer L., Csernoch L., Pusztahelyi T., Emri T., Posci I., Salvenmoser W., Marx F. (2005). Antifungal protein PAF severely affects the integrity of the plasma membrane of *Aspergillus nidulans* and induces an apoptosis-like phenotype. Antimicrob. Agents Chemother..

